# The public health approach to violence reduction: A process evaluation of a UK inner-city serious youth violence strategy

**DOI:** 10.1016/j.puhe.2026.106260

**Published:** 2026-06

**Authors:** J.T. Oha, J. Wills, F. Morrow, T. Mills, S. Sykes

**Affiliations:** College of Health and Life Sciences, London South Bank University, 103 Borough Road, London, SE1 0AA, UK

**Keywords:** Public health, Youth violence, Violence prevention, Violence reduction, Process evaluation

## Abstract

**Objectives:**

To examine the opportunities and challenges of applying a public health approach to violence prevention within a ten-year youth violence reduction strategy in an inner-London region.

**Study design:**

Multi method study within a process evaluation.

**Methods:**

The study investigated: (a) the influence of a public health approach on strategy design through documentary analysis and two focus groups with local government staff and community leaders (n = 11); (b) contextual factors shaping engagement with the approach via two actor-mapping workshops (n = 18) and narrative interviews (n = 7); (c) implementation of the public health approach in strategy delivery through four focus groups and one interview with staff and community leaders (n = 19), and interviews with participating young people or their carers (n = 10); and (d) a review of key performance indicators and a focus group discussion (n = 9).

**Results:**

The public health approach supported an evidence-based strategy but was inconsistently articulated and understood, creating implementation challenges. Despite a complex violence-reduction landscape, the voluntary and community sector sustained productive relationships with the strategy. Data challenges included limited outcome measurement, with mostly quantitative indicators largely focused on tracking intervention activity.

**Conclusion:**

While the public health approach remains the dominant model for violence prevention, challenges persist in shared understanding, inter-agency collaboration, and data generation and sharing. Findings highlight the need to engage with local complexity through a place- and asset-based approach to youth violence narratives.

## 1 Introduction

Over 40% of homicides occur amongst 15–29-year-olds, totalling 193,000 annually.^1^ The World Health Organization (WHO) define youth violence as violence amongst unrelated 10-29-year-olds who may know each other.^1^ Irrespective of fatality, violence has far-reaching consequences for health, impacting victims, perpetrators, bystanders, families, and the community, alongside law enforcement, health and social care, education, welfare and local government services.^2^ The United Nations (UN) Convention of the Rights of the Child list youth violence as a priority area for national governments.^3^

In the UK, serious youth violence is a significant concern. In 2018, The UK Government published its Serious Violence Strategy, responding to increases in knife and gun crime, serious violence and homicides.^4^ To support strategy delivery, the UK Home Office launched multiple initiatives, including Violence Reduction Units, to bring together partners in a multi-agency response, building local capacity to address the root causes of serious violence in twenty of the worst affected areas across England and Wales.^5^

**Fig. 1:**
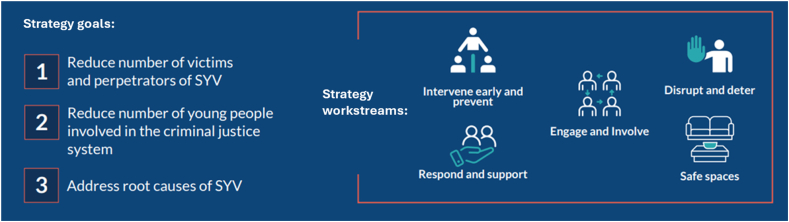
A UK inner-city strategy to reduce serious youth violence∗, ‘Lambeth Made Safer’. Overarching strategy goals (left) and five multi-agency delivery workstreams (right). ∗Serious youth violence is defined locally as ‘interpersonal violence involving 10- to 25-year-olds that may involve weapons or physical harm’.

Lambeth local government, an inner-city region in London, launched a ten-year strategy in 2020 termed “Lambeth Made Safer “that aims to: (1) Reduce serious youth violence victims and perpetrators; (2) Lower youth criminal justice involvement; and (3) Tackle root causes of serious youth violence. It is implemented via a multi-agency approach that includes schools, police, NHS, residents and community groups, faith-based organisations. There are five workstreams shown in Fig. 1 that include varied activities: Intervene Early and Prevent (e.g., parenting support); Disrupt and Deter (e.g., stop and search); Respond and Support (e.g., hospital outreach); Engage and Involve (e.g., arts programmes); and Safe Spaces (e.g., video surveillance) delivering interventions at contextual, primary, secondary, and tertiary levels.

**Fig. 2:**
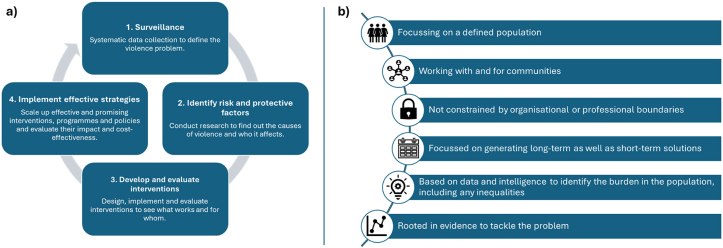
Models of a Public Health Approach (PHA) to Violence Reduction (VR). **A)** World Health Organization (WHO) four-step model of a PHA to VR **b)** UK Violence Reduction Units adaptation of the WHO model of a PHA to VR.

The WHO define a public health approach to violence reduction using a typology of violence, and a socioecological framework^6^ (Fig. 2). This approach prioritises early intervention and holistic forms of support aimed at preventing violence at its source, and has underpinned the establishment of more than 20 Violence Reduction Units across England and Wales. The approach looks at violence as a preventable consequence of a range of factors, such as adverse early life experiences or harmful social or community experiences. Consequently, it broadens the remit of violence prevention beyond traditional law-enforcement responses to encompass health services, social care, education, youth provision and voluntary, community, faith and social enterprise (VCFSE) sectors.

Although the public health approach is viewed as the leading violence prevention model, implementation challenges have been reported in different contexts.^7^, ^8^, ^9^ These include a lack of clarity on what constitutes the core characteristics of a public health approach;^8^ that the public health approach to violence reduction is not a single entity, but rather an assorted set of principles, practices, and discourses;^9^, ^10^, ^11^ a paucity of evidence around how to implement a programme of evidence-based interventions across primary to tertiary categories,^10^ and a lack of consistency about public health approach implementation.^12^

This paper is part of a wider process evaluation of the development and early implementation of the ‘Lambeth Made Safer’ strategy. Guided by the UK Medical Research Council's framework for evaluating complex interventions,^13^ we sought to understand the implementation process, mechanisms of impact and strategy context. A public health approach was said to underpin the strategy and so the evaluation investigated how well it was offering a way of working to address serious youth violence by: facilitating multi-agency collaboration that brings together all organisations and professionals whose remit and concern includes the reduction of violence; encouraging interventions based on the best available evidence and data; and encouraging community engagement.

## 2 Methods

**Fig. 3:**
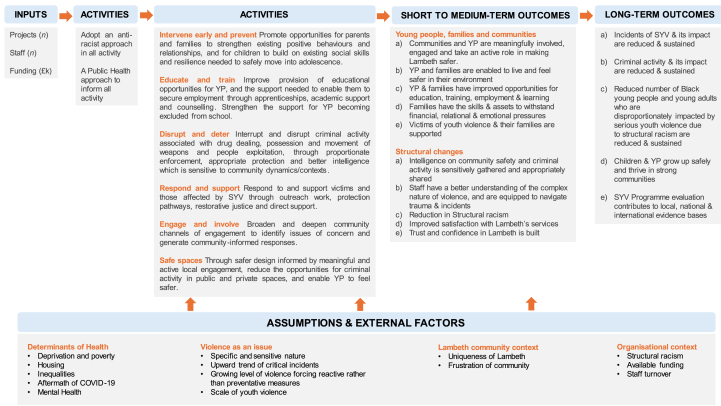
A logic model of the Lambeth Made Safer (LMS) strategy. Developed with key LMS stakeholders and public contributors (Public and Patient Involvement and Engagement (PPIE) representatives), over a series of co-production workshops.

Drawing on the UK Medical Research Council framework for process evaluations,^13^ a programme theory for this evaluation was developed. This is shown in a logic model (Fig. 3) which represents the ways in which interventions/activities were expected to lead to violence reduction outcomes and the theory of change underpinning the strategy. The public health approach, by focusing on understanding root causes of serious youth violence and using available data and intelligence including community insights, could contribute to these changes.

The wider evaluation focused on exploring the implementation of the strategy. This paper reports only on the implementation of the public health approach which was explored across all of the data sources (see Table 1). Coproduction workshops were facilitated by the evaluation team, where stakeholders and public contributors (Public and Patient Involvement and Engagement (PPIE) representatives), described the strategy's short to medium and longer-term outcomes. The research team was supported by the PPIE panel (n = 10) and two peer researchers who collected data and helped to conduct analysis of the interviews with young people who took part in two of the activities.

**Table 1:** Data collection sources.

Objective	Data collection method	Data source/participants
To review how the public health approach is understood and shapes strategy development	Desktop documentary analysis Focus groups x2	Strategy documents (n = 25) Local government staff and community leaders (n = 11)
To understand the context for strategy implementation and multi-sector collaborative partnerships	Actor mapping workshops x 2 Narrative interviews	Local government staff and community leaders (n = 18) Longstanding residents, local government staff, community leaders (n = 7)
To review the evidence base for the commissioned activities/interventions	Desktop evidence review	(1) The Youth Endowment Fund (YEF) Toolkit and LMS-aligned YEF reports; (2) Recently published or pre-print peer-reviewed and grey literature that did not yet appear in the YEF Toolkit, prioritising UK-based evidence; and (3) Grey literature relevant to the local context of Lambeth, published post-March 2019, the publication date of the last substantive review.
To explore how the public health approach was being implemented	Focus groups x2 Semi structured interviews	Local government staff, and community leaders (n = 19) Young people participating in activities/interventions or their carers (n = 10)
To understand how data informed the development of the strategy for serious youth violence	Desktop review of key performance indicators Focus group	38 publicly available KPIs Local government staff and community leaders (n = 9)

We recruited forty-five participants using opportunity sampling:^14^ local government staff and community leaders (n = 28) were invited to participate via the strategy's programme manager; young people participating in activities/interventions or their carers through community leaders (n = 10); and residents via local government community leads (n = 7). To widen participation, we offered online and in-person options for in focus groups and interviews.

We employed multiple methods of data collection described in Table 1. Questions designed to explore the public health approach were included in all evaluation data collection tools. The range of methods provided comprehensive and nuanced insight from stakeholder perspectives and strategy documentation, enabling data triangulation on the ways in which the public health approach was included in the strategy conceptualisation and implementation.

Qualitative data were analysed thematically in Delve software.^15^, ^16^ Coders met regularly to ensure inter-coder reliability and rigour.^17^ We adopted a hybrid inductive/deductive approach to analysis,^18^ informed by our programme theory (Fig. 3). Inductive analysis enabled a more open exploration of the data, allowing related but non-predefined aspects of the public health approach and its operational context to be identified. Deductive analysis was based on a coding framework derived from the WHO and Violence Reduction Unit frameworks (see Fig. 2).

## 3 Results

This process evaluation of a serious youth violence strategy at its mid-point, illustrates challenges and opportunities of maintaining a public health approach through design to delivery. Findings are organised into three themes: strategy development, implementation and context.

### 3.1 Strategy development

The public policy and political climate for action on serious youth violence resulted in local government support and resourcing for a long-term strategy. Described as “… a need to be seen to be doing something” (Focus group 1), this was underscored by incidents of violence alongside calls from the VCFSE groups. One participant commented:

[…] there had been some incidents and there was a real voice from the community […] saying what’s happening, what are we going to do […] something needed to happen. […] also as professionals we acknowledged that […] *Focus group 1*.

There was a clear commitment when writing the strategy that it would be based on a public health approach and this was evidenced in documents and focus groups. Place-based intelligence from consultations, needs assessments, police and education were used to identify risk factors, priority groups and issues, such as Black young people, parents and families. Participants believed that evidence of effective interventions was used during the development stages including both national evidence but also locally derived evidence and an understanding of what works locally. There was a strong commitment in the documentary data and focus groups that the strategy should be driven by or with communities.

### 3.2 Strategy implementation

The public health approach was less evident during strategy delivery, as the process shifted from planning the overarching vision to enacting its mission through executing projects:

There’s lots of people, very busy operationally, […] doing some really productive work, but it’s not quite coordinated or joined up […] it does feel like it’s happening because operational workers and managers are stretching their load, rather than there being a coherent, corporate drive. *Focus group 3*.

Two challenges for operationalising the public health approach were: ensuring a contemporary review of intervention evidence given the evolving nature of serious youth violence, such as the influence of organised criminal groups and changing weapon use; and establishing robust outcome measures.

A public health approach focuses on the identification of root causes of serious youth violence. Outcome measures that are essential for assessing implementation impact and scaling effective programmes would need to be both short term and immediate but also longer term and evidence reductions in violence. The strategy employed a range of Key Performance Indicators (n = 38): whilst some of these were useful for measuring outcomes across workstreams, many were quantitative tracking intervention activity (e.g., numbers of people completing interventions). This led to gaps in measuring strategy-level outcomes, particularly in the long-term and at societal level.

Whilst the strategy's public health approach emphasised the use of local intelligence, a change in statutory partner data capture systems led to reduced local government access to key fields (e.g., location and setting of violent incidents). Also, altered crime metrics (e.g., knife crime definitions) disrupted trend analyses of violence reduction outcomes, whilst incomplete or overly broad demographic data (e.g., gender, ethnicity) hampered understanding of which young people were most at risk and how best to protect them, and intervention delivery staff.

The location is a big one. We can map it, but we have no idea if it took place on a bus, outside a shop, and when you’re protecting the young people you need that detail unless it comes from the police, and that’s a big thing and that’s why it’s really hard to draw context out of it, out of a lot of numbers. *Interviewee #19*

A public health approach emphasises community engagement but maintaining the connectivity was described as difficult. There was also a focus on improving outcomes by listening and jointly designing interventions. Interviews with young people taking part in some of the interventions or their carers revealed that long-term programmes enabled those participating to build trusting relationships with peers and staff, supporting a sense of both physical and psychological safety.

### 3.3 Strategy context

The public health approach emphasises cross-sector partnership. Actor-mapping workshops (see Table 1) were held firstly to identify the key organisations, groups and individuals influencing violence reduction in the area; and secondly to discuss relationships between actors and the strategy, as well as opportunities and gaps for productive engagement. They identified a large and complex violence reduction network of over eighty actors, reflecting both the scale of serious youth violence and ambitious responses. Despite its scale and complexity, participants noted assets of strong partnerships, particularly with VCFSE groups, whilst opportunities with faith groups and strategic boards were underused:

[…] representatives of statutory agencies don’t easily work with faith groups. They get horribly 1980s PC (politically correct) about working with religious groups. Unlike with the counterterrorism Prevent programme to a degree […] where you need to engage. *Actor mapping workshop*

Societal-level actors such as local government counter-terrorism boards, showed the fewest productive relationships (12.8%, n = 5/39). Although national justice and interior ministries (e.g., UK Home Office) were mapped as part of the violence reduction system, neither were seen as strongly connected to strategy operations. Questionnaire data revealed strategy operations were viewed as active and productive (median scores 7/10, n = 18).

Narrative interviews revealed local assets, enablers and barriers rooted in place, which influenced strategy implementation. There was a keenness to understand the root causes of serious youth violence coupled with frustration over undervalued cultural knowledge. At a societal level, there were efforts to broaden discussions to include girls and shift away from negative narratives to ‘what works’: this was described alongside feelings of resignation at persistent systemic and institutional issues such as delayed therapeutic support for Black youth.

We will often get asked for media comments […] we’d rather focus on […] what we know really works and makes a difference […] than getting pulled into these kinds of conversations which are framed in a negative. *Interviewee #7*

## 4 Discussion

This evaluation echoes previously reported implementation limitations of the WHO model.^6^ Four challenges to the adoption of a public health approach to serious youth violence were identified: (a) An unclear understanding of how to apply a public health approach to the specific issue of serious youth violence locally; (b) Gaps in available surveillance data from statutory partners affecting the ability to identify local risk and protective factors; (c) A lack of robust qualitative outcome data to evaluate long-term and societal strategy-level impacts; and (d) Multi-agency collaboration where there are a large number of violence reduction actors and a wide range of contextual factors operating as both barriers and enablers to implementation. The evaluation identifies an important but previously overlooked area in violence reduction: recognising how context shapes effective, asset and place-based narratives about serious youth violence. Instead of emphasising risks or risk factors, these narratives focus on the strengths of a defined group in a specific location and how those strengths contribute to reducing violence^19^ and place-based narratives about serious youth violence.

Much of the public health approach violence reduction literature refers to socioecology as a framework for developing comprehensive responses across individual, relationship, community and societal levels.^6^ Strategy documents and participant responses reflected this, where the intention to address upstream protective and risk factors and work with violence reduction stakeholders across socioecological levels was clear. Attention to systemic causes of violence aligns with recommendations around maintaining focus on macro-level drivers of violence,^19^ including the use of local disproportionality data which in this locality, was weighted towards the disproportionate impact of serious youth violence amongst Black youth.^20^

Although Walsh and colleagues describe a lack of studies characterising the core characteristics of a public health approach to violence reduction,^8^one of the acknowledged consistent features is the need to understand the problem. This involves collecting and generating data on the nature and scope of serious youth violence, and evaluating the effectiveness of violence reduction efforts.^7^^,^^9^ This evaluation highlighted local challenges associated with the quality of evidence due to changing data capture systems, altered crime metrics impacting trend analyses and incomplete demographic data affecting risk assessment. Moreover, stakeholders highlighted opportunities to improve measurement through cross-departmental data sharing (e.g., housing, social care, youth offending, education), where internal networking and dissemination of strategy impact data were viewed as opportunities to build trust and demonstrate the value of shared data through illustrating progress to partners.

The conflation of short, medium and long-term outcomes, a feature highlighted in guidance to disaggregate outcomes,^21^ hindered implementation of strategic ambitions underpinned by the public health approach and may have contributed to the strategy's focus on monitoring intervention-level activity over strategy outcomes. Outcome monitoring largely required qualitative data, such as that collected through community engagement,^9^ whereas existing measures were largely quantitative. Data challenges were compounded by serious youth violence being a fast-moving field, where the nature of violence and therefore how to measure it, continues to evolve.

Complex local environments with concurrent barriers and facilitators to strategy implementation, present a challenge for effective strategy delivery. Historically, local context has been under accounted for in violence reduction efforts.^19^ Engaging young people, families, and communities can help to understand and harness facilitators as assets, creating an enabling environment sensitive to local inequalities, contextual differences, and risk factors. This translates as an opportunity to create an asset- and place-based serious youth violence narrative, harnessing factors such as community pride, a drive to understand root causes of violence, and success stories from outcomes data, to support effective strategy implementation.

Multi-agency collaboration is central to a public health approach to violence reduction given the scale and complexity of violence reduction networks at local, city and national levels. This is reflected in the CAPRICORN framework of serious youth violence prevention which describes a ‘whole system multi-agency approach that is place-based’.^22^

A challenge for implementing a public health approach which delivers evidence-based, comprehensive, context-relevant, sustainable strategies for serious youth violence is that appropriate data is required to inform intervention commissioning and impact assessment. Additionally, consensus amongst stakeholders is needed to prioritise target groups and issues, with community engagement an important factor in accounting for the local environment, ensuring strategies are context-specific.^9^

### 4.1 Limitations

Existing literature on violence reduction strategy implementation and the adoption of a public health approach is largely descriptive. While this this academic-local government partnership was able to explore the ‘how’ of implementation, it was within a limited time frame and with a relatively small sample size. A history of external organisations undertaking scrutiny exercises in the locality as well as the sensitive nature of serious youth violence, resulted in scepticism and understandable issues of trust during data collection. The nature of rapid evaluation meant it was neither possible to build the necessary relationships with VCSFE groups, nor to navigate safeguarding arrangements required to engage with those involved in tertiary interventions. Despite attempts to mitigate this through the evaluation co-production process, by recruiting through trusted gatekeepers and offering both online and face-to-face data collection options, there was only limited recruitment of those participating in the interventions.

Implementing such strategies with fidelity requires ‘adaptability’ (organisational ability to understand the levels of change needed to successfully replicate programmes), and ‘compatibility’ (contextual appropriateness of a programme of interventions to address well-defined problems).^23^ Effectively transferring evidence-based programmes across settings is thus a complicated long-term process, but this study has added a real-world example of how the public health approach illuminates some practicalities of implementation.^11^, ^24^

### 4.2 Conclusion

A public health approach to a serious youth violence strategy demonstrated the value of public health principles in identifying upstream risk and protective factors and fostering community engagement through community groups, particularly amongst global majority populations.

However, challenges still remained in fully operationalising a public health approach to serious youth violence, due to an identified need to: co-develop locally understood serious youth violence public health frameworks; use evidence in all its forms including published literature, stakeholder insights and local learning, revisiting initial strategy theory to understand context-specific mechanisms of strategy impact; invest in resources to strengthen strategy outcome measurement; and work towards a whole system, multi-agency approach that is place-based and adopts an asset-based approach to communicating serious youth violence narratives to foster an enabling environment for strategy implementation.

## Ethical statement

The evaluation was carried out in accordance with the Declaration of Helsinki. Ethical approval was secured from London South Bank University Ethics Committee (ETH2324-0140, ETH2324-0252 and ETH2425-0068). All participants gave formal written and/or recorded verbal informed consent to participate and for their data to be used.

## Data sharing

Given the sensitive nature of the evaluation topic and challenges of fully anonymising qualitative data, raw data will not be made available to maintain participant confidentiality. Additional outputs are available here: https://phirst.nihr.ac.uk/evaluations/lambeth-serious-youth-violence/. The evaluation protocol is available here: https://fundingawards.nihr.ac.uk/award/NIHR135964.

## Funding

10.13039/501100000272National Institute for Health and Care Research (NIHR) NIHR131568/NIHR135540. For open access, authors applied a Creative Commons Attribution (CC BY) licence to any Author Accepted Manuscript version arising. The views expressed are those of the author(s) and not necessarily those of the NIHR or the Department of Health and Social Care.

## Declaration of competing interest

The authors declare that the National Institute for Health and Care Research (NIHR) (NIHR131568/NIHR135540) funded this research and publication. Research was conducted in the absence of commercial or financial relationships that could be construed as a potential conflict of interest.
